# Genetic predisposition may not improve prediction of cardiac surgery-associated acute kidney injury

**DOI:** 10.3389/fgene.2023.1094908

**Published:** 2023-04-13

**Authors:** Nicholas J. Douville, Daniel B. Larach, Adam Lewis, Lisa Bastarache, Anita Pandit, Jing He, Michael Heung, Michael Mathis, Jonathan P. Wanderer, Sachin Kheterpal, Ida Surakka, Miklos D. Kertai

**Affiliations:** ^1^ Department of Anesthesiology, University of Michigan Health System, Ann Arbor, MI, United States; ^2^ Center for Computational Medicine and Bioinformatics, University of Michigan Health System, Ann Arbor, MI, United States; ^3^ Michigan Integrated Center for Health Analytics and Medical Prediction, Institute for Healthcare Policy and Innovation, University of Michigan, Ann Arbor, MI, United States; ^4^ Department of Anesthesiology, Vanderbilt University Medical Center, Nashville, TN, United States; ^5^ Department of Biomedical Informatics, Vanderbilt University Medical Center, Nashville, TN, United States; ^6^ Center for Statistical Genetics and Precision Health Initiative, University of Michigan, Ann Arbor, MI, United States; ^7^ Division of Nephrology, Department of Internal Medicine, University of Michigan, Ann Arbor, MI, United States; ^8^ Division of Cardiovascular Medicine, Department of Internal Medicine, University of Michigan, Ann Arbor, MI, United States

**Keywords:** perioperative genomics, acute kidney injury, cardiac surgery-associated acute kidney injury, precision medicine and genomics, anesthesiology [H02.403.066], polygenic risk score (PRS)

## Abstract

**Background:** The recent integration of genomic data with electronic health records has enabled large scale genomic studies on a variety of perioperative complications, yet genome-wide association studies on acute kidney injury have been limited in size or confounded by composite outcomes. Genome-wide association studies can be leveraged to create a polygenic risk score which can then be integrated with traditional clinical risk factors to better predict postoperative complications, like acute kidney injury.

**Methods:** Using integrated genetic data from two academic biorepositories, we conduct a genome-wide association study on cardiac surgery-associated acute kidney injury. Next, we develop a polygenic risk score and test the predictive utility within regressions controlling for age, gender, principal components, preoperative serum creatinine, and a range of patient, clinical, and procedural risk factors. Finally, we estimate additive variant heritability using genetic mixed models.

**Results:** Among 1,014 qualifying procedures at Vanderbilt University Medical Center and 478 at Michigan Medicine, 348 (34.3%) and 121 (25.3%) developed AKI, respectively. No variants exceeded genome-wide significance (*p* < 5 × 10^−8^) threshold, however, six previously unreported variants exceeded the suggestive threshold (*p* < 1 × 10^−6^). Notable variants detected include: 1) rs74637005, located in the exonic region of *NFU1* and 2) rs17438465, located between *EVX1* and *HIBADH*. We failed to replicate variants from prior unbiased studies of post-surgical acute kidney injury. Polygenic risk was not significantly associated with post-surgical acute kidney injury in any of the models, however, case duration (aOR = 1.002, 95% CI 1.000–1.003, *p* = 0.013), diabetes mellitus (aOR = 2.025, 95% CI 1.320–3.103, *p* = 0.001), and valvular disease (aOR = 0.558, 95% CI 0.372–0.835, *p* = 0.005) were significant in the full model.

**Conclusion:** Polygenic risk score was not significantly associated with cardiac surgery-associated acute kidney injury and acute kidney injury may have a low heritability in this population. These results suggest that susceptibility is only minimally influenced by baseline genetic predisposition and that clinical risk factors, some of which are modifiable, may play a more influential role in predicting this complication. The overall impact of genetics in overall risk for cardiac surgery-associated acute kidney injury may be small compared to clinical risk factors.

## Introduction

Up to 30% of cardiac surgeries are complicated by postoperative acute kidney injury (AKI) (O’Neal et al., 2016), which dramatically increases hospital and late mortality, even in patients with subsequent renal recovery. (Engoren et al., 2014). Cardiac surgery can expose patients to a unique set of physiologic stressors, including cardiopulmonary bypass, blood product transfusion, vasopressors, and aortic cross-clamping. Consequently, cardiac surgery-associated AKI has overlap, but also potentially unique mechanisms, when compared with other subtypes of AKI (Frank et al., 2012). Furthermore, the increased rate of AKI following cardiac surgery and the detailed data collection inherent to the perioperative setting may provide an opportunity to better study and understand AKI.

Advances in the availability and analysis of genetic information enable genetic risk to be incorporated into the patient-specific risk profile for multiple postoperative complications (Kolek et al., 2015; Dudbridge, 2016; Torkamani et al., 2018; Douville et al., 2020; Hornsby et al., 2020; Kertai et al., 2021; Kullo et al., 2022). Notably, polygenic risk score (PRS) improved the accuracy for predicting atrial fibrillation following cardiac surgery-associated (Kolek et al., 2015; Kertai et al., 2021) and myocardial injury after non-cardiac surgery (Douville et al., 2020) when compared to predictive models utilizing traditional clinical risk factors. While PRS have been developed to predict chronic kidney disease (Yu et al., 2021; Khan et al., 2022), the heritability of acute kidney injury has not been well characterized (Larach et al., 2018) and cardiac surgery-associated AKI is further complicated by dynamic intraoperative variables including surgical technique, intraoperative hypotension, and anemia (Vives et al., 2019; Mathis et al., 2020). However, since cardiac surgery-associated AKI cannot be entirely explained by known risk factors, it is possible that AKI has a genetic component. Prior genome-wide association studies (GWAS) on cardiac surgery-associated AKI have revealed only a few significant associations (Stafford-Smith et al., 2015; Zhao et al., 2017; Westphal et al., 2019). Furthermore, these studies were limited by small sample size, heterogeneous population (Zhao et al., 2017), or composite outcome (Westphal et al., 2019), leaving open the possibility that the genetic contribution to cardiac surgery-associated AKI could be uncovered in a large study involving standardized phenotype across two healthcare systems (Larach et al., 2018). If this were the case, polygenic risk scoring could be used to better predict and ideally prevent this outcome. Alternatively, it may be that the genetic contribution to cardiac surgery-associated AKI is limited.

Our primary objective is to perform a GWAS on AKI following cardiac surgery using integrated genetic data from two academic biorepositories. We will leverage the results of our GWAS to create a PRS to quantify the combined contribution from hundreds of thousands of genetic variants (Khera et al., 2018), which can be integrated with traditional clinical risk factors in an attempt to better predict cardiac surgery-associated AKI. The secondary objective is to test the predictive utility of a novel PRS for predicting acute kidney injury following cardiac surgery.

## Materials and methods

### Study design

This multi-center genome-wide association study followed the recommendations of Strengthening the Reporting of Genetic Association Studies (Little, 2009). Study outcomes, data collection, and statistical analyses were established *a priori* and presented at a multidisciplinary peer-review forum (4 February 2021) prior to data access (Multicenter Perioperative Outcomes Group, 2017). The study was approved by the Institutional Review Boards at both Vanderbilt (191829) and Michigan (HUM00126162). As no patient care interventions were made through conducting the study, patient consent was waived.

### Study population

Inclusion criteria for the study were all patients who underwent cardiac surgery at Vanderbilt University Medical Center (“Vanderbilt”; Nashville, TN, United States) and Michigan Medicine of the University of Michigan (“Michigan”; Ann Arbor, MI, United States) and had genotype data available. For patients with more than one cardiac procedure, only the first case was considered. Patients were excluded if they had severe preoperative renal dysfunction or preoperative renal failure^1^ or were missing primary anesthesia CPT code data. Additional exclusion criteria were 1) American Society of Anesthesiologists Physical Status Classification VI; 2) procedure duration <45 min; 3) subsequent surgery within 7 days without documentation of a postoperative serum creatinine between cases; 4) implausible body mass index;^2^ 5) surgery classified as outpatient or 23-hour-admission; 6) or missing primary anesthesia base CPT code data.

### Primary outcome

The primary outcome was Stage 1, 2, or 3 kidney injury as defined by the *Kidney Disease: Improving Global Outcomes* (KDIGO) practice guidelines within 7 days following surgery or discharge (whichever occurred first) (Kellum et al., 2012). Baseline preoperative serum creatinine was established using the closest value to the time of surgery (within 1-year), as previously described.^3^ (Mathis et al., 2020) The diagnostic criteria for the primary outcome relied on serum creatinine or receipt of dialysis, and did not incorporate urine output, which was not consistently recorded for the entire postoperative surveillance window in our dataset.

### Data collection

#### Genotype

Genetic data was obtained from Vanderbilt’s DNA biobank (BioVU) and Michigan Medicine’s Michigan Genomics Initiative (MGI). BioVU enrolled surgical patients beginning in 2008, while MGI began in 2012. Study participants provided written informed consent for obtaining genetic data, and protocols were reviewed and approved by the Institutional Review Board (Douville et al., 2020). Due to the retrospective design of the specific-study and the use of deidentified data, the need for additional informed patient consent was waived by both boards. Full details and characterization of genotype for both cohorts can be found in Supplementary Appendix SA1.

#### Electronic health record data

Donated blood samples were coded and linked to full EHR data. We extracted a limited dataset of relevant clinical outcomes for each patient meeting inclusion criteria from the Multicenter Perioperative Outcomes Group (MPOG) database. Methods for data input, validation, storage, and extraction within the MPOG consortium have been described elsewhere (Multicenter Perioperative Outcomes Group, 2017) and utilized in previous studies (Bender et al., 2015; Aziz et al., 2016; Lee et al., 2017). Extracted variables included patient demographics (age, sex), preoperative characteristics [body mass index, creatinine, hemoglobin, and Elixhauser comorbidity measures (Elixhauser et al., 1998)] and procedural factors (case duration) that have been previously identified as risk factors for cardiac surgery-associated AKI (Kheterpal et al., 2009). Time-stamped serum creatinine values were obtained from each patient and curated to define the primary outcome.

#### Statistical analysis

Clinical variables were characterized in both the Vanderbilt and Michigan Medicine cohorts. Perioperative characteristics were summarized using means and standard deviations for normally distributed continuous covariates, medians and interquartile range (IQR) for non-normally distributed continuous variables and counts and percentages for categorical covariates.

#### Genome-wide association study (GWAS)

We performed GWAS using PLINK 1.9 (PLINK, 2022) for all genotyped and imputed markers (BioVU 11,292,079; MGI 10,642,058; Meta-analysis 6,291,460) assuming an additive inheritance model (homozygote major allele vs. heterozygote vs. homozygote minor allele). Overall effect of candidate SNPs were assessed using the effect size estimates and standard errors across each site. Meta-analysis was performed using a fixed-effects meta-analysis approach implemented in METAL (Metal—Meta Analysis Helper; Center for Statistical Genetics, 2017) to assess each candidate SNP and prioritize candidate genes.

Genome-wide significance threshold (*p* < 5 × 10^−8^) was selected *a priori* for identification of novel loci and a lower *suggestive* threshold (*p* < 1 × 10^−6^). SNPs identified at the suggestive threshold (*p* < 1 × 10^−6^) were subsequently analyzed in three logistic regression models. Model 1, controlled for 1) age, 2) gender, and 3) the first four principal components (PCs). In Model 2, preoperative serum creatinine was added to the variables in Model 1. Finally, in Model 3, we controlled for the full range of patient, clinical, and procedure related risk factors. Performance of each model was quantified *via* area under the receiver operating characteristic curve (c-statistic)^4^. For multivariable logistic regressions, *p* < 0.05 was considered statistically significant. Analyses were performed using R version 4.0.0. (R Foundation for Statistical Computing, Vienna, Austria) unless stated otherwise.

#### Polygenic risk scoring (PRS)

For polygenic risk calculation and validation, the MGI and the BioVU populations were treated as independent datasets for *base* and *target*, respectively (Chatterjee et al., 2016; Choi et al., 2020). Descriptive statistics generated from the MGI cohort were used to calculate PRS for all patients within the BioVU cohort *via* the method of Bayesian regression and continuous shrinkage priors, which has been demonstrated to have greater predictive accuracy across a range of complex diseases and quantitative traits when compared to alternative methods for calculating polygenic risk (Ge et al., 2019). In this method, posterior effect sizes of single nucleotide polymorphisms are inferred using GWAS summary statistics (derived from the MGI cohort) and an external linkage disequilibrium reference panel (United Kingdom Biobank - European population) using default Stradwerman-Berger prior^5^. The Polygenic Risk Score for Cardiac Surgery-Associated Acute Kidney Injury (PRS_AKI_) for each patient in the BioVU population was then converted into a standardized score with mean 0 and standard deviation of 1 to facilitate interpretation. The three regressions were then repeated in just the validation cohort with PRS_AKI_ included as the exposure variable.

#### Heritability model

Next, we estimated the additive SNP heritability in the BioVU cohort using genetic mixed models, as implemented in the Genome-wide Complex Trait Analysis (GCTA) pack (Yang et al., 2011). Heritability was defined as the proportion of cardiac surgery-associated AKI liability attributable to additive common genetic variation. The GCTA method uses a mixed model in conjunction with a genetic relationship matrix (GRM) of pairs of samples based on 840,964 SNPs to estimate the proportion of variance explained by genetic similarity between individuals. GCTA provides a lower limit of the variance that can be theoretically explained by common SNPs. In our study, the estimated heritability was transformed from a liability scale to an observed scale assuming the incidence of cardiac surgery-associated AKI was 4.5%. The GCTA analyses were adjusted for age, gender and 4 PCs.

#### Power analysis

We used a convenience sample of available cardiac cases with available genotype data at Vanderbilt University Medical Center and Michigan Medicine. *Post-hoc* power analysis was performed to calculate *allelic frequency* and *genotype relative risk* pairings that our study was powered to detect at a statistical power of 0.80 using the University of Michigan Center for Statistical Genetics Power Calculator (GAS Power Calculator, 2017). The number of cases (478) and controls (1,014) from the combined cohort were used in this calculation. Genome-wide significance level (*p* < 5 × 10^−8^), prevalence of 0.08, (Hoste et al., 2008), and *additive* disease model were *a priori* assumptions used in the power calculations. At an allelic frequency of 0.01 (the minimum included in our analysis); variants would have to increase the relative risk of disease by a factor of 6 to be detected with statistical power of 0.80. The study is better powered to detect more common genetic variants with moderate effect size (for example, a variant with allelic frequency 25%, increasing the relative risk of the disease by 1.7x could be detected with statistical power of 0.80).

## Results

### Patient population—Baseline characteristics

The Vanderbilt (BioVU) population had a median age of 62.9 (IQR 55.4–72.0) and was 67% male. Median case duration was 188.7 min (IQR 109.3–254.0). The Michigan Medicine (MGI) cohort had a median age 63.5 years (IQR 55.0–72.0), and was 69% male. Median case duration was 310.0 min (IQR 230.2–407.5). In the Michigan Medicine Cohort, the majority had hypertension (71.8%) and valvular disease (79.7%). Population characteristics for both BioVU and MGI cohorts are shown in Table 1.

**TABLE 1:** Baseline and clinical characteristics of the study population.

Characteristics	Vanderbilt cohort, *n* = 1014	Michigan cohort, *n* = 478	*p* value
Age, years	62.9 [55.4–72.0]	63.5 [55.0–72.0]	0.475
Male sex	680 (67.1%)	329 (68.8%)	0.727
Body mass index, kg/m^2^	29.4 [25.2–32.7]	29.7 [26.0–33.2]	0.044
Preoperative creatinine value, mg/dL	1.14 [0.87–1.24]	0.94 [0.81–1.10]	0.214
Case duration, min	188.7 [109.3–254.0]	310.0 [230.2–407.5]	<0.001
Elixhauser comorbidities			
Chronic pulmonary disease	190 (18.7%)	144 (30.1%)	<0.001
Congestive heart failure	211 (20.8%)	215 (45.0%)	<0.001
Diabetes mellitus	142 (14.0%)	123 (25.7%)	<0.001
Hypertension	346 (34.1%)	343 (71.8%)	<0.001
Valvular disease	209 (20.6%)	381 (79.7%)	<0.001

### Outcome table

**FIGURE 1:**
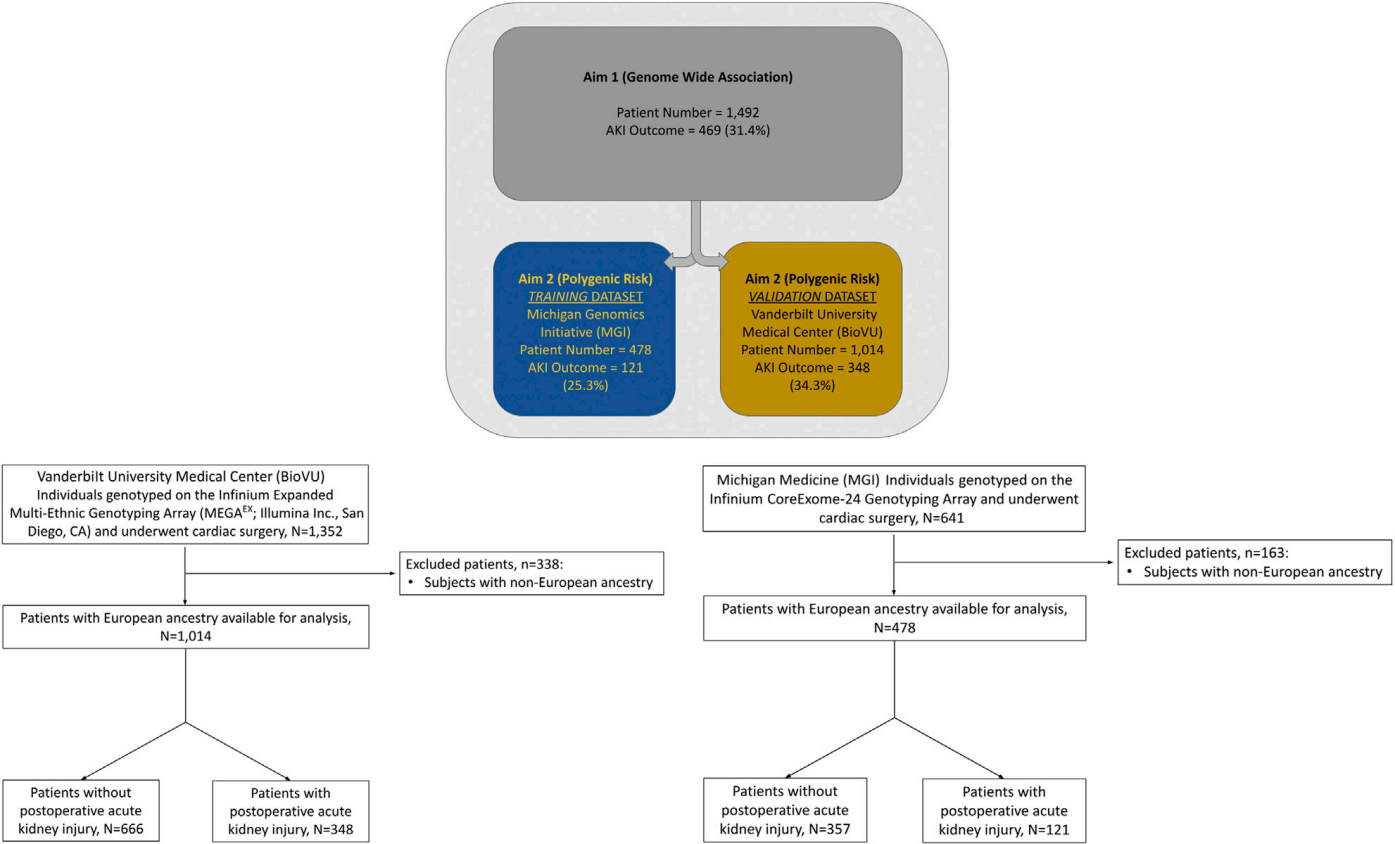
Cohort derivation and outcome characterization.

Among 1,014 qualifying procedures at Vanderbilt University Medical Center and 478 at Michigan Medicine, 348 (34.3%) and 121 (25.3%) developed cardiac surgery-associated AKI, respectively (Figure 1). Cardiac surgery-associated AKI developed more frequently following longer procedures, and in patients with underlying comorbidities: chronic pulmonary disease and diabetes mellitus (Table 2). Most patients in both cohorts developed KDIGO, Stage 1 AKI. Additional details regarding the breakdown by KDIGO stage can be found in Supplementary Table S1.

**TABLE 2:** Characteristics of the study population developing cardiac surgery-associated acute kidney injury compared to population not developing cardiac surgery-associated acute kidney injury.

	Vanderbilt cohort			Michigan cohort		
Characteristics	Yes AKI, *n* = 348	No AKI, *n* = 666	*p* value	Yes AKI, *n* = 121	No AKI, *n* = 357	*p* value
Age, years	64.7 [57.8–73.2]	62.0 [54.3–71.3]	0.002	64.0 [57.0–73.0]	63.0 [54.0–71.0]	0.916
Male sex	235 (66.8%)	445 (67.5%)	0.819	82 (67.8%)	247 (69.2%)	0.821
Body mass index, kg/m^2^	30.2 [25.5–33.5]	28.9 [24.9–32.3]	0.425	30.82 [26.7–34.5]	29.57 [25.9–32.9]	0.304
Preoperative creatinine value, mg/dL	1.3 [0.94–1.4100]	1.06 [0.83–1.16]	2.23 × 10^−10^	1.00 [0.90–1.20]	0.93 [0.8–1.10]	0.156
Case duration, min	182.3 [81.5–247.5]	184.3 [105.25–250.50]	0.584	333.0 [265–418]	299.0 [227–399]	0.021
Elixhauser comorbidities						
Chronic pulmonary disease	80 (16.5%)	110 (23.0%)	0.013	47 (38.8%)	97 (27.2%)	0.022
Congestive heart failure	92 (26.4%)	119 (17.9%)	0.002	61 (50.4%)	154 (43.1%)	0.171
Diabetes mellitus	72 (20.7%)	70 (10.5%)	1.29 × 10^−5^	47 (38.8%)	76 (21.3%)	2.72 × 10^−4^
Hypertension	141 (40.5%)	205 (30.8%)	0.002	95 (78.5%)	248 (69.5%)	0.062
Valvular disease	73 (17.7%)	136 (16.4%)	0.835	93 (76.9%)	288 (80.7%)	0.363

### Genome-wide association study (GWAS) results

Meta-analysis was performed on 6,291,460 SNPs. Among those: 5,364,338 SNPs had R^2^ ≥ 0.8 in both datasets and 927,122 SNPs had R^2^ < 0.8 in at least one dataset. No single nucleotide polymorphisms (SNP) exceeded our pre-determined threshold for genome-wide significance (*p* < 5 × 10^−8^) on any of the three logistic regression models. In the basic regression model, Model 1, which controlled for age, gender, and first four principal components, two SNPs were identified at the suggestive threshold: rs12421245 (*p* = 3.86 × 10^−7^) and rs73131342 (*p* = 8.76 × 10^−7^). The genomic inflation factor (λ) for Model 1 was 0.986. When preoperative serum creatinine was also controlled for (in addition to age, gender, and first four principal components) (Model 2), rs12421245 remained below the suggestive threshold (*p* = 3.96 × 10^−7^), rs73131342 was no longer below the suggestive threshold (*p* = 3.72 × 10^−6^), and rs3847598 decreased to below the suggestive threshold (*p* = 7.55 × 10^−7^). In Model 3, the logistic regression controlling for a full group of clinical covariates, rs12421245 (*p* = 9.81 × 10^−7^) remained suggestive. Additionally, three new SNPs were identified: rs113741905 (*p* = 1.47 × 10^−7^), rs74637005 (*p* = 9.58 × 10^−7^), and rs17438465 (*p* = 8.74 × 10^−7^). Full GWAS results for each of the three models can be found in Table 3. There was no significant heterogeneity between the two centers in any of the reported SNPs (Supplementary Table S2). Manhattan Plots and Quantile-Quantile (QQ) Plots for each of the three Models (meta-analysis) are shown in Supplementary Information (Supplementary Figure S1).

**TABLE 3:** Logistic regression analysis of genetic predictors of cardiac surgery-associated acute kidney injury in the study populations.

						Vanderbilt cohort				Michigan cohort						Combined
						EAF				EAF						
Chr		SNP	POS	Test allele	Gene symbol	No AKI	Yes AKI	OR (95% CI)	P	No AKI	Yes AKI	OR (95% CI)	P	Meta-OR	SE (OR)	Meta-P
Model 1: Adjusted for age, gender, 4 principal components																
11		rs12421245	6965384	A	ZNF215	0.10	0.16	1.80 (1.35–2.40)	7.21 × 10^−5^	0.13	0.21	1.99 (1.31–3.06)	0.001	1.35	1.06	3.86 × 10^−7^
20		rs73131342	58586654	T	CDH26	0.02	0.06	3.30 (1.86–5.83)	4.21 × 10^−5^	0.02	0.06	3.05 (1.37–6.79)	0.006	1.86	1.13	8.76 × 10^−7^
Model 2: Adjusted for age, gender, 4 principal components, and preoperative serum creatinine																
11		rs12421245	6965384	A	ZNF215	0.10	0.16	1.76 (1.31–2.37)	1.72 × 10^−4^	0.13	0.21	2.20 (1.41–3.41)	4.65 × 10^−4^	1.88	1.13	3.96 × 10^−7^
11		rs3847598	33966695	A	LMO2 | CAPRIN1	0.82	0.76	0.63 (0.50–0.80)	1.13 × 10^−4^	0.84	0.74	0.52 (0.35–0.78)	1.40 × 10^−3^	0.60	1.11	7.55 × 10^−7^
Model 3: Adjusted for age, gender, 4 principal components, preoperative serum creatinine, body mass index, Elixhauser comorbidity measures (chronic pulmonary disease, congestive heart failure, diabetes mellitus, hypertension, valvular disease), and case duration																
1		rs113741905	17811179	A	RCC2 | ARHGEF10L	0.20	0.15	0.57 (0.41–0.78)	5.79 × 10^−4^	0.25	0.13	0.44 (0.30–0.65)	4.29 × 10^−5^	0.51	1.14	1.47 × 10^−7^
2		rs74637005	69650730	A	NFU1	0.03	0.06	3.26 (1.89–5.63)	2.15 × 10^−5^	0.02	0.05	3.35 (1.27–8.86)	1.46 × 10^−2^	3.28	1.27	9.58 × 10^−7^
7		rs17438465	27365380	T	EVX1 | HIBADH	0.40	0.30	0.60 (0.47–0.75)	1.46 × 10^−5^	0.40	0.31	0.67 (0.48–0.93)	1.70 × 10^−2^	0.62	1.10	8.74 × 10^−7^
11		rs12421245	6965384	A	ZNF215	0.10	0.16	1.82 (1.32–2.51)	2.35 × 10^−4^	0.13	0.21	2.11 (1.35–3.31)	1.06 × 10^−3^	1.92	1.14	9.81 × 10^−7^

AKI, acute kidney injury; Chr, chromosome; CI, confidence interval; Meta, meta-analysis; OR, odds ratio; POS, position; SE, standard error; SNP, single-nucleotide polymorphism; EAF, effect allele frequency.

Next, we attempted to determine the gene associated with each SNP identified in the three models. We characterized the function of each variant exceeding the suggestive threshold (Supplementary Table S3). For non-exonic SNPs, the closest gene by chromosome position was selected. Genes identified using this methodology were: *ZNF215*, *CDH26*, *LMO2/CAPRIN1*, *RCC2/ARHGEF10L*, *NFU1*, and *EVX1/HIBADH* (Table 3).

### Attempted replication of prior GWAS findings

We failed to replicate SNPs from prior unbiased studies of post-surgical acute kidney injury (Supplementary Table S4) (Westphal et al.; Stafford-Smith et al., 2015; Zhao et al., 2017).

### Polygenic risk score

**FIGURE 2:**
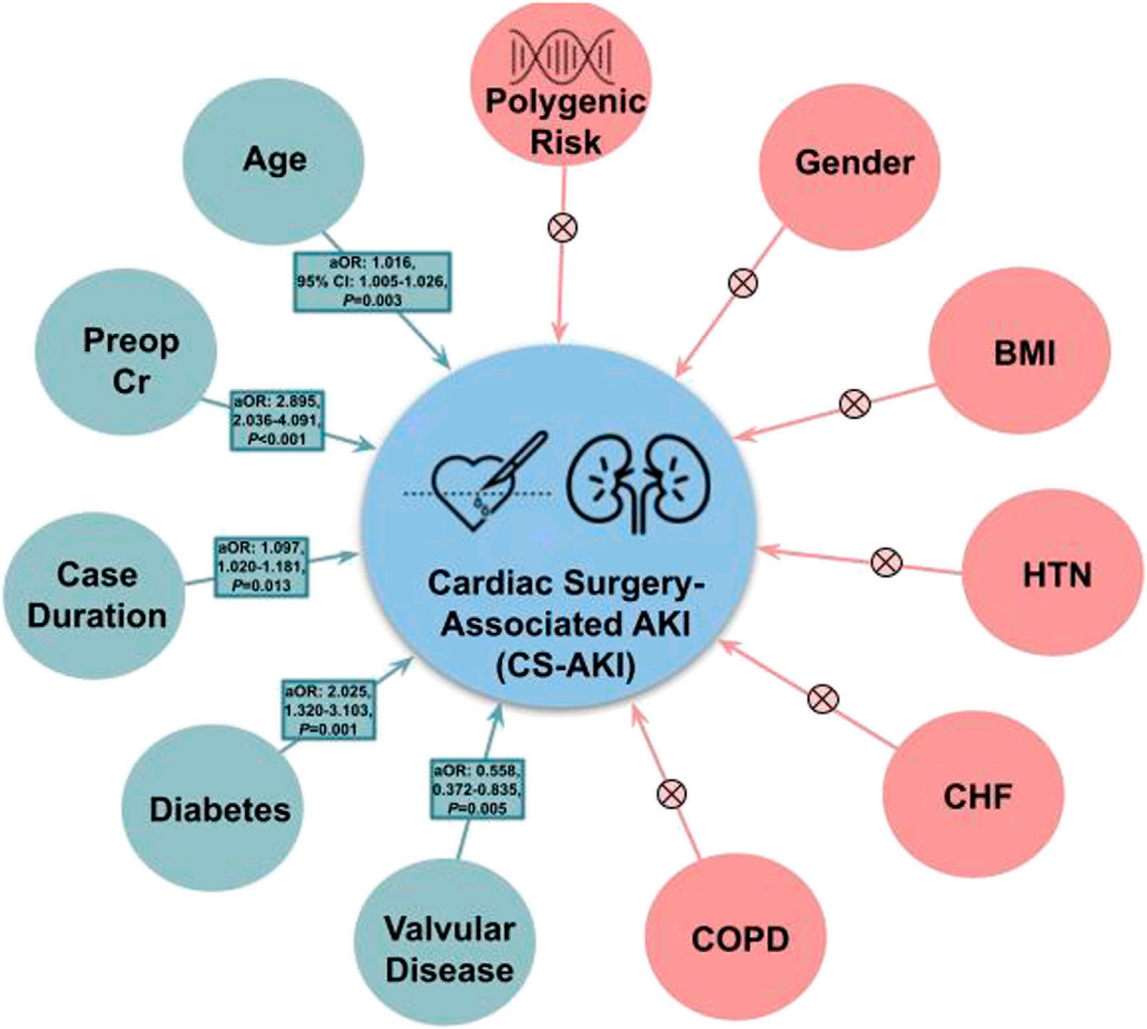
Clinical factors associated with acute kidney injury following cardiac surgery. Factors found to be associated with Cardiac Surgery-Associated Acute Kidney Injury are shown in green with corresponding adjusted odds ratio (aOR), 95% Confidence Interval (95% CI), and *p*-value. Those factors that were not significant are shown in red with a corresponding “X”. Units: Age (years), Preoperative Serum Creatine (mg/dL), BMI (kg/m^2^), case duration (hours). Abbreviations: 95% CI, 95% confidence interval; aOR, adjusted odds ratio; BMI, body mass index; CS-AKI, cardiac surgery-associated acute kidney injury; CHF, congestive heart failure; COPD, chronic obstructive pulmonary disease; HTN, hypertension; Preop Cr, preoperative serum creatinine.

When included as a covariate within Model 1, Model 2, and Model 3 of the validation cohort (BioVU), polygenic risk was not significantly associated with post-surgical acute kidney injury (Table 4) (*p*-value ranged from 0.117 in Model 3 to 0.215 in Model 1). Age was significant in all 3 models and preoperative serum creatinine was significant in both models that it was included as a covariate. In addition to age and preoperative serum creatinine; case duration in minutes (aOR = 1.002, 95% CI 1.000–1.003, *p* = 0.013), diabetes mellitus (aOR = 2.025, 95% CI 1.320–3.103, *p* = 0.001), and valvular disease (aOR = 0.558, 95% CI 0.372–0.835, *p* = 0.005) were included in Model 3 (the full clinical model). Clinical factors associated with cardiac surgery-associated AKI (and those that could not be shown to have a significant association) can be visualized in Figure 2. Full results of the three Models with PRS_AKI_ included can be found in Table 4. The discrimination was 0.573 (95% CI: 0.537–0.610) for Model 1; 0.661 (95% CI: 0.626–0.697) for Model 2; and 0.681 (95% CI: 0.646–0.715) for Model 3.

**TABLE 4:** Logistic regression analysis of genetic predictors of cardiac surgery-associated acute kidney injury in the BioVU study populations with inclusion of Polygenic Risk Score.

Variable	aOR	95% CI		*p*-value
Model 1: Adjusted for age, gender, 4 principal components				
Age (years)	1.016	1.005	1.026	0.003
Female gender	0.965	0.730	1.274	0.801
Polygenic risk score	0.000	0.000	Inf	0.215
Model 2: Adjusted for age, gender, 4 principal components, and preoperative serum creatinine				
Age (years)	1.012	1.002	1.023	0.022
Female gender	1.122	0.839	1.499	0.436
Preoperative serum creatinine (mg/dL)	2.956	2.094	4.141	0.000
Polygenic sisk score	0.000	0.000	Inf	0.148
Model 3: Adjusted for age, gender, 4 principal components, preoperative serum creatinine, body mass index, Elixhauser comorbidity measures (chronic pulmonary disease, congestive heart failure, diabetes mellitus, hypertension, valvular disease), and case duration				
Age (years)	1.014	1.003	1.025	0.011
Female gender	1.182	0.877	1.592	0.270
Preoperative serum creatinine (mg/dL)	2.895	2.036	4.091	0.000
Body mass index (BMI) kg/m^2^	0.997	0.988	1.007	0.601
Case duration (hours)	1.097	1.020	1.181	0.013
Chronic pulmonary disease	1.172	0.792	1.735	0.427
Congestive heart failure	1.392	0.946	2.049	0.093
Diabetes mellitus	2.025	1.320	3.103	0.001
Hypertension	1.184	0.830	1.688	0.351
Valvular disease	0.558	0.372	0.835	0.005
Polygenic risk score	0.000	0.000	Inf	0.117

Model 1: c-statistic = 0.573 (95% CI: 0.537–0.610); Model 2: c-statistic = 0.661 (95% CI: 0.626–0.697); and Model 3: c-statistic = 0.681 (95% CI: 0.646–0.715).

### Heritability

The heritability of cardiac surgery-associated AKI was calculated to be <0.001 with standard error of 0.378.

## Discussion

In the largest genome-wide association study on acute kidney injury following cardiac surgery, we were not able to replicate previously identified variants and genes to prior studies. Furthermore, we found that a novel PRS was not significantly associated with cardiac surgery-associated AKI. This contrasts with other post-surgical complications, like atrial fibrillation and myocardial injury, where polygenic risk improves risk prediction models (Kolek et al., 2015; Douville et al., 2020; Kertai et al., 2021). Our results suggest that susceptibility to cardiac surgery-associated AKI may only be minimally influenced by baseline genetic predisposition, contrasting with chronic kidney disease (Wuttke and Köttgen, 2016). Furthermore, clinical risk factors like age in years (aOR 1.014, 95% CI 1.003–1.025, *p* = 0.011), preoperative serum creatinine (aOR 2.895, 95% CI 2.036–4.091, *p* < 0.001), case duration in hours (aOR 1.097, 95% CI 1.020–1.181, *p* = 0.013), and diabetes mellitus (aOR 2.025, 95% CI 1.320–3.103, *p* = 0.001) have greater utility in a model with c-statistic of 0.681 (95% CI: 0.646–0.715).

### Candidate genes

Our study failed to identify SNPs exceeding the pre-specified genome-wide significance level (*p* < 5 × 10^−8^). However, using the *a priori* defined lower *suggestive* threshold (*p* < 1 × 10^−6^) identified 6 previously unreported SNPs which may be associated with acute kidney injury following cardiac surgery (Supplementary Table S3). The SNP rs74637005 was located in the exonic region of *NFU1*, a gene previously shown to be associated with both calcium levels (Sakaue et al., 2021) and elevation in metabolic biomarkers (Martin et al., 2021). Three SNPs were located in intergenic regions (rs3847598, rs113741905, and rs17438465) and two SNPs were intronic (rs12421245 and rs73131342) (Supplementary Table S3). Notably, rs17438465 is located between *EVX1* and *HIBADH*, genes that have been associated with nephrolithiasis and cardiovascular disease (Howles et al., 2019; Kichaev et al., 2019). No genes identified by proximity-based candidate gene analysis had previously been reported to be associated with acute or chronic renal disease.

Furthermore, SNPs exceeding suggestive threshold were interrogated using the HaploReg tool (HaploReg v4.1) (Ward and Kellis, 2012) with key findings highlighted in Supplementary Table S3. Notably, rs3847598 is predicted to be bound by GATA2, a protein already described in AKI (Yu et al., 2017), and rs12421245 is annotated as expression quantitative trait loci of ZNF215 in different tissues. Additionally, rs73131342 alters the Pax8 and Sox motifs. Pax8 is notably a nephric-lineage transcription factor required for renal organogenesis (Tong et al., 2009) potentially involved in regeneration and recovery after acute renal injury (Zivotic et al., 2022) and Sox activation is an early transcriptional response to acute kidney injury (Kumar et al., 2015).

These findings should be considered hypothesis generating and require additional replication and validation.

### Comparison to prior studies

We failed to replicate any of the variants previously reported to be associated with cardiac surgery-associated acute kidney injury. Differences in phenotype potentially account for this finding. Our study focused on the narrow phenotype of acute kidney injury following cardiac surgery, whereas, the RIPHeart-Study assessed a broad composite of complications occurring following cardiac surgery (although they did adjust for each individual complication) (Westphal et al.). The one SNP in the RIPHeart-Study exceeding genome-wide significance (rs78064607 located in *PHLPP2*) for AKI was notably not available for inclusion in our meta-analysis and no SNPs in close linkage disequilibrium could be identified to provide a suitable approximation.

We had a similar incidence of cardiac surgery-associated AKI as defined by the KDIGO criteria (31.4% compared with 33.0% in the combined PEGASUS and CATHGEN cohorts) compared to the prior GWAS in cardiac surgery-associated AKI (Stafford-Smith et al., 2015). The GWAS was performed using the continuous variable, percent rise in serum creatinine from baseline level within 10-day following surgery, compared to our KDIGO-based outcome. Zhao and colleagues reported an AKI incidence of 35.8% in the discovery cohort from the Translational Research Investigating Biomarkers Endpoints in AKI (TRIBE) cardiac surgery study and 14.7% (206 cases and 1,406 controls) in the replication cohort (CABG Genomics and Brigham’s Cardiac Surgery Study sub-cohorts) (Zhao et al., 2017). This study defined AKI cases as patients with at least a 0.3-mg/dL or 50% increase in serum creatinine from baseline for at least two consecutive days and non-AKI controls as patients with an increase in serum creatinine not exceeding 25% relative to baseline (Zhao et al., 2017).

The majority of patients in our study developed KDIGO Stage 1 AKI (89% of all AKI in the BioVU cohort and 88% in MGI; Supplementary Table S1). Restricting the analysis to only the patients developing Stage 2 or Stage 3 AKI may reveal a stronger genetic linkage, however, this small sample size precluded this analysis in our 2-center cohort. Furthermore, the incidence in our cohort exceeds that reported in lower risk cardiac surgery cohorts (Hu et al., 2016), suggesting the presence of high-risk cases in the dataset (for example, those involving the aorta, requiring hypothermic circulatory arrest, or inserting ventricular assist devices). In these high-risk cases, the genetic component may contribute very little to predictive accuracy, but for low risk cases (for example, non-emergent, isolated coronary artery bypass graft or isolated valve) genetic susceptibility may play a greater role. Further studies, restricted to lower risk cardiac cases, may reveal populations where genetics exerts larger impact on overall risk profile.

### Study strengths and limitations

We assess genetic associations between a large number of genetic polymorphisms (BioVU 11,292,079; MGI 10,642,058; Meta-analysis 6,291,460) across a large number of patients (1,492) and surgical procedures. This contrasts to prior genetic studies in AKI which have largely employed the candidate gene approach (Larach et al., 2018). Additionally, we expanded the breadth of our genetic analyses from GWAS to develop a PRS, which quantifies the combined contribution from hundreds of thousands of genetic variants, each of which may individually have a small effect. This single number was then integrated with traditional clinical risk factors in an attempt to better predict AKI.

A major strength of the study was the collaboration between two academic biorepositories (BioVU and MGI). This provided the largest possible cohort for GWAS (BioVU + MGI), and allowed validation of PRS in an independent dataset (MGI = Base Dataset, BioVU = Target Dataset). Our meta-analysis had minimal genomic inflation (λ = 0.986). Meta-analysis of suggestive SNPs also confirmed the directionality of the effect. Another strength of our study was the use of a validated data collection method (covariates) combined with the standardized KDIGO outcome. The major limitation of our study is that we are underpowered to detect the effect of low-frequency variants, exerting only a moderate impact on the relative risk of AKI. Despite this limitation, we assess novel associations and attempt to replicate previously described associations in two independent biobanks with well-curated phenotypic and procedural details. We selected *base* (MGI) and *target* (BioVU) cohorts based upon ready availability of the additional clinical features within BioVU that we incorporated into the full clinical model (Model 3). As prediction accuracy of PRS depends mostly on the heritability of the analyzed traits, the number of SNPs, and the size of the discovery sample; our PRS is inherently limited based upon the sample size of our base MGI cohort.

Another inherent limitation of our dataset is the lack of genetic diversity in both biobanks. While data within both BioVU and MGI reflect the patient population served by both institutions and lack of ancestral diversity is commonly encountered problem across many biobanks (Zhou et al., 2019), integration of our data with global biobanks composed of individuals from more diverse ancestries will enable even more equitable, impactful research.

While our evidence suggests that there is limited heritability of postsurgical AKI in the cardiac surgery population, this study only interrogated common genetic variants (minor allelic frequency >1%). Thus, the possibility remains that rare variants contribute risk (or even protection) for some cardiac surgery patients. Our heritability calculation is likely limited by smaller than needed effective sample size, limiting interpretability and generalizability to larger cohorts (Neale Lab, 2017). In GWAS studies with small effective sample sizes (i.e., below 5,000–10,000), statistical artifacts can result in near zero heritability estimates, even in phenotypes with known heritability. Our small sample size and large standard error likely limits the utility of this heritability analysis and further studies with larger sample size are required to better assess heritability of cardiac surgery-associated AKI. Finally, index event bias may be impacting discovery of biologically relevant genetic risk variants, although this is less relevant for risk prediction. Despite these limitations, our results suggest that polygenic risk scores based upon common variants likely have limited predictive utility.

## Conclusion

This large multi-center GWAS of cardiac surgery-associated AKI failed to identify any genome-wide significant associations. We found 6 variants associated with acute kidney injury at the suggestive threshold (*p* < 1 × 10^−6^). Among these, rs74637005 is located in the exonic region of *NFU1*, a gene previously shown to be associated with both calcium levels (Sakaue et al., 2021) and elevation in metabolic biomarkers (Martin et al., 2021) and rs17438465 is located between *EVX1* and *HIBADH*, genes that have been associated with nephrolithiasis and cardiovascular disease (Howles et al., 2019; Kichaev et al., 2019). Polygenic risk was not significantly associated with cardiac surgery-associated AKI, and our findings also indicated a low heritability for cardiac surgery-associated AKI. These results suggest that susceptibility to cardiac surgery-associated AKI is minimally influenced by baseline genetic predisposition and clinical risk factors, some of which are modifiable, may play a more influential role in predicting this postoperative complication. While it is possible that 1) larger sample sizes, 2) analysis of sub-populations (for example, low-risk cardiac surgery or patients developing more severe stage AKI), or 3) assessment of low-frequency variants (since variants with an allelic frequency <1% were not included in the PRS) may demonstrate a higher degree of genetic influence in predicting this complication—the overall impact of genetics in overall risk for cardiac surgery-associated AKI may be small compared to clinical risk factors.

## Data Availability

The raw data supporting the conclusion of this article will be made available by the authors, without undue reservation.

## Footnotes

1 Preoperative renal dysfunction defined as: baseline eGFR <15 ml/min/1.73 m^2^, hemodialysis dependent [based on the presence of prior anesthesia CPT code for dialysis access or International Classification of Diseases (ICD) codes for end-stage renal disease] preoperatively, or had preoperative AKI (based upon relative serum creatinine increase of >50% or absolute serum creatinine increase of >0.3 mg/dL) during the 7 days prior to surgery.

2 Implausible body mass index values defined as <10 or >80 kg/m^2^.

3 Cardiac surgery-associated AKI was defined using the most recent preoperative (baseline) serum creatinine values that were collected up to 365 days before surgery and all available serum creatinine values that were measured within 7 days after surgery or until discharge, whichever came first. The diagnosis of cardiac surgery-associated AKI was made if there was a 50% or greater serum creatinine increase in the first 7 postoperative days, or a 0.3 mg/dL increase detected using a rolling 54-h window starting at anesthesia end time and ending 7 days later in the postoperative period. The 54-h rolling window was selected to add 3 h buffer on each side of the usual 48-h window to account for minor variations in serum creatinine blood draw and laboratory reporting times.

4 Interpretation of the c-statistic: 0.5 or less for a poor model, over 0.7 for a good model, over 0.8 for a strong model, 1.0 for a perfect model (Hosmer and Lemeshow, 2000).

5 A = 1, B = 0.5.
