# Supplementary material for: Genetic predisposition may not improve prediction of cardiac surgery-associated acute kidney injury
Source: Front Genet. 2023 Apr 13;14:1094908. doi: 10.3389/fgene.2023.1094908 (PMC10133500; doi:10.3389/fgene.2023.1094908)
Supplement: Supplementary file 1 [file DataSheet1.pdf]

Supplementary Table 1. Outcomes Table

**Vanderbilt University Medical Center**		Number of Patients	
No AKI	Stage 0	666	666
AKI	Stage 1	311	
	Stage 2	28	
	Stage 3	9	348
**Michigan Medicine**		Number of Patients	
No AKI	Stage 0	357	357
AKI	Stage 1	106	
	Stage 2	14	
	Stage 3	1	121

Supplementary Table 2. Heterogeneity Between Biobanks

Chr	*Pos*	*SNP*	*Meta-analysis P-value*	*Heterogeneity I^2*	*Cochran's Q-Test*	*Heterogeneity P-value*
**Model I**						
11	6965384	rs12421245	3.86×10^{-7}	0.0	0.164	0.686
20	58586654	rs73131342	8.76×10^{-7}	0.0	0.024	0.877
**Model II**						
11	6965384	rs12421245	3.96×10^{-7}	0.0	0.654	0.419
11	33966695	rs3847598	7.55×10^{-7}	0.0	0.639	0.424
**Model III**						
1	17811179	rs113741905	1.47×10^{-7}	0.0	0.947	0.330
2	69650730	rs74637005	9.58×10^{-7}	0.0	0.002	0.961
7	27365380	rs17438465	8.74×10^{-7}	0.0	0.301	0.583
11	6965384	rs12421245	9.81×10^{-7}	0.0	0.283	0.595

Legend: I^2 statistic which ranges between 0-1, 1 being highly heterogeneous and 0 not at all.
SNP = single nucleotide polymorphism

Supplementary Information, Table 3. Function and Nearest Gene for Variants Exceeding the Suggestive Threshold

SNP ID	Effect Allele	Alternative Allele	MAF	OR	*P*-Value	Function	Gene
rs12421245	A	G	0.122	1.859	$3.86x10^{-7}$	intronic	ZNF215
rs73131342	T	C	0.032	3.210	$8.76x10^{-7}$	intronic	CDH26
rs3847598	C	A	0.197	1.667	$7.55x10^{-7}$	intergenic	LMO2; CAPRIN1
rs113741905	A	G	0.190	0.510	$1.47x10^{-7}$	intergenic	RCC2; ARHGEF10L
rs74637005	A	G	0.033	3.283	$9.58x10^{-7}$	exonic	NFU1
rs17438465	T	G	0.363	0.618	$8.74x10^{-7}$	intergenic	EVX1;HIBADH

Legend: Additional Information from *HaploReg v4.1*:
rs12421245 - eQTL: ZNF215; Motifs altered: Ets_disc7, Mxi1_disc1, Pitx2, Znf143_disc3
rs73131342 - Motifs changed: Pax-8, Sox
rs3847598 - Enhancer histone marks: BLD; DNAse: BLD; Proteins bound: GATA2, MAFK, TAL1; Motifs changed: MAF, NF-Y
rs113741905 - Motifs changed: ZID, p300
rs74637005 - Motifs changed: Maf, Rad21; GENCODE genes: NFU1
rs17438465 - Motifs changed: Barx1, Barx2, Dbx2, En-1_3, Esx1, Gbx1, Gbx2, HNF1_5, Hlxb9, Hoxa7_2, Hoxb3, Hoxb7, Ik-1_1, Ik-3, NF-kappaB, Nkx6-1_3, Pax-6_3, Phox2a, Pou2f2, Pou3f4, Prrx1, RORalpha1_2, STAT_known5, Vax2

Supplementary Information, Table 4. Attempted Replication of Prior GWAS findings

		Discovery cohort Stafford-Smith *et al.*, 2016			*Replication Cohort* BioVU/MGI, 2022		
**SNPID**	**Gene**	**MAF**	**OR**	**P-value**	**MAF**	**OR**	**P-value**
rs1488349	GRM7, LMCD1	0.02/0.01	29.71	5.41×10^{-10}	0.018	0.632420979	0.165
rs28619003	BBS9	0.08/0.10	15.58	6.51×10^{-8}	0.120	1.146828149	0.284
rs13317787	GRM7|LMCD1-AS1	0.03/0.02	21.56	5.35×10^{-7}	0.033	0.815054742	0.478
rs10262995	BBS9	0.09/0.10	14.33	2.24×10^{-7}	0.114	1.144536784	0.291
		Westphal *et al.*, 2019			BioVU/MGI, 2022		
rs78064607	PHLPP2	0.01	50	3.77×10^{-8}	*	*	*
rs189437718	Metazoa_SRP, ST3GAL1-DT	0.01	20	3.60×10^{-7}	*	*	*
rs72654815	EIF4G3	0.03	9	6.79×10^{-7}	0.025	1.023778301	0.930
rs77876049	CLMP, HSPA8	0.05	4	9.04×10^{-6}	0.042	0.937817417	0.755
		Zhao *et a*l, 2016			BiovVU/MGI, 2022		
rs62341639	APOL1 regulator, IRF2	0.18/0.14	0.64	2.48×10^{-7}	*	*	*
rs62341657	APOL1 regulator, IRF2	0.18/0.14	0.65	3.26×10^{-7}	*	*	*
rs9617814	AKI-related gene TBX1	0.22/0.20	0.70	3.81×10^{-6}	0.238	0.89511775	0.231

rs10854554	AKI-related gene TBX1	0.19/0.17	0.67	$6.53x10^{-7}$	0.205	0.92108773	0.4030
		Larach *et al*, 2022 (non-cardiac)			BiovVU/MGI, 2022		
rs975593	D21S2088E; LINC01689	0.115	1.353	$6.62x10^{-7}$	0.109	0.95113431	0.689
rs2255595	ADARB2	0.561	0.804	$8.62x10^{-7}$	0.561	0.92524196	0.363
rs143469518	PRR15L; CDK5RAP3	0.025	1.770	$8.46x10^{-7}$	0.027	1.29641142	0.274
rs2069295	SP2-AS1	0.024	1.894	$4.84x10^{-7}$	0.027	1.293	0.276
rs117284771	CDK5RAP3	0.025	1.901	$3.69x10^{-7}$	0.028	1.264	0.321

* The SNPS: s78064607, rs189437718 were not included/available in the meta-analysis. Since they are rare (MAF<1%), no SNPs in close linkage disequilibrium could be identified to provide a suitable approximation. The SNPs: rs62341639 and rs62341657 were not available in our dataset.

Supplementary Information, Figure 1. **Manhattan Plots and Quantile-Quantile (QQ) Plots**

A. Model 1: adjusted for age, gender, 4 principal components

Manhattan Plot

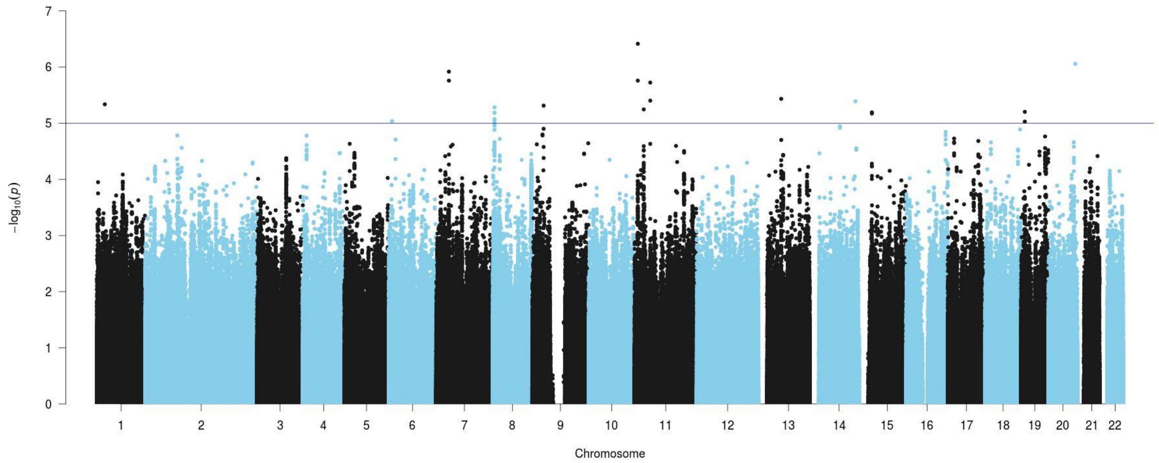

QQ-Plot

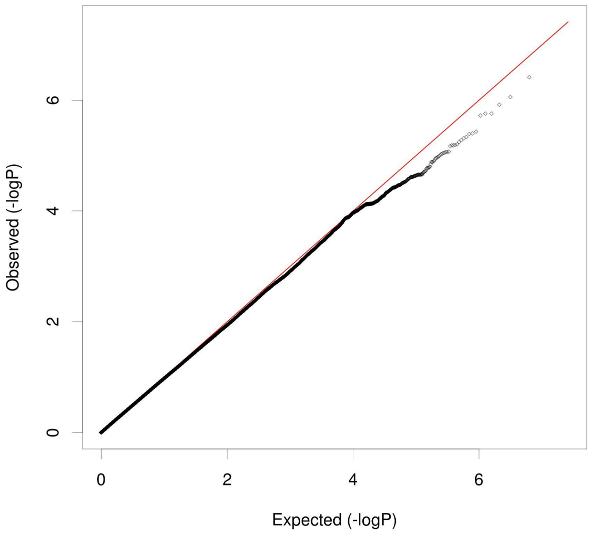

B. <u>Model 2</u>: adjusted for age, gender, 4 principal components, and preoperative serum creatinine

Manhattan Plot

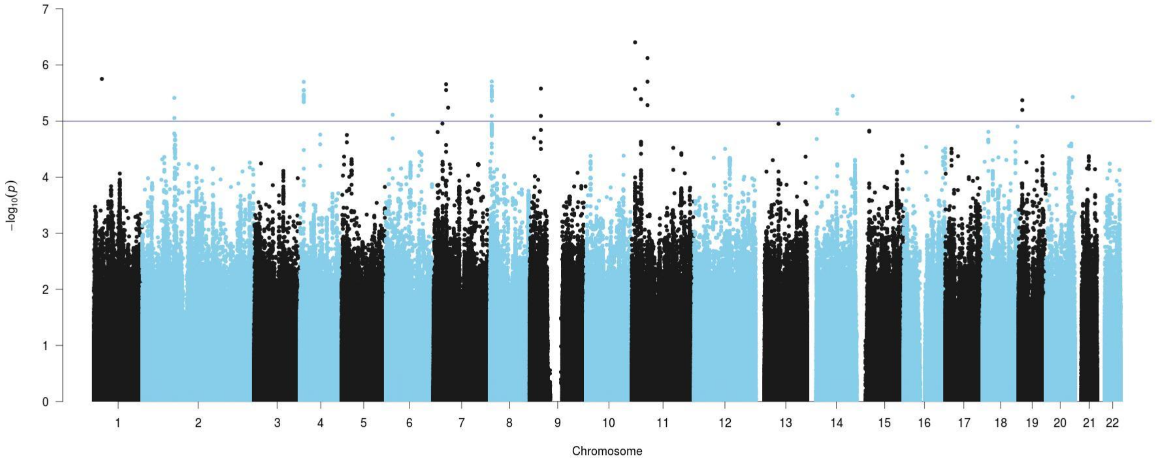

QQ-Plot

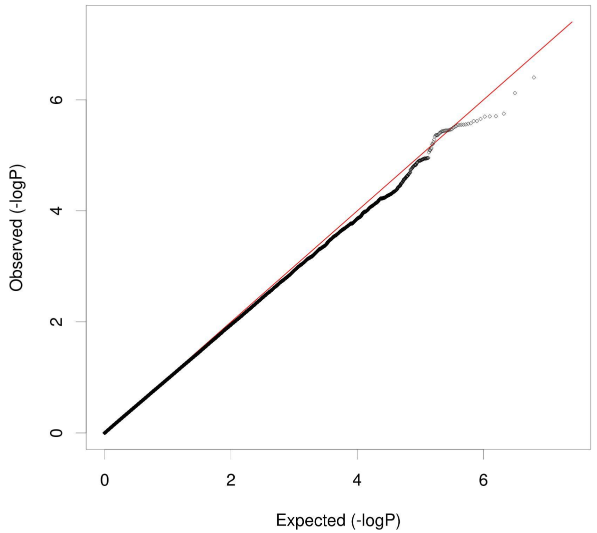

C. <u>Model 3</u>: adjusted for age, gender, 4 principal components, preoperative serum creatinine, body mass index, Elixhauser comorbidity measures, and case duration

Manhattan Plot

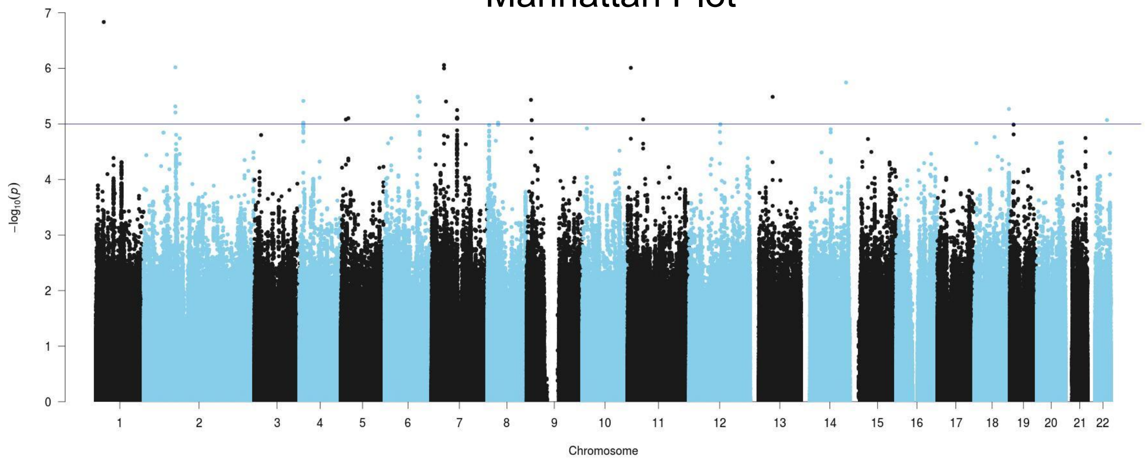

QQ-Plot

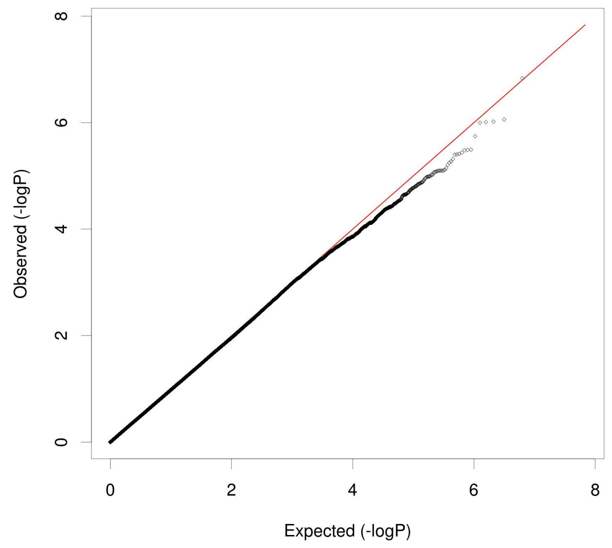

Appendix 1: Genotyping Protocol and Characterization of Genetic Data

Vanderbilt Cohort

Subjects were genotyped using DNA extracted from discarded plasma samples with the Illumina Infinium® Expanded Multi-Ethnic Genotyping Array ($MEGA^{EX}$, Illumina Inc., San Diego, CA, USA). Imputation of the Vanderbilt $MEGA^{EX}$ genotyped dataset was performed using the Minimac4 and 1000 Genomes Phase 3 v5 with the Michigan Imputation Server. Variants with $R^2 \geq 0.3$ and minor allele frequency (MAF) $\geq$ 1% were selected, resulting in more than 21.1 million overall imputed variants available and 11.7 million imputed variants available for the AKI GWAS with White European ancestry after quality control and filtering.

Michigan Cohort

Subjects were genotyped using Illumina Infinium® CoreExome-24 bead Arrays (Illumina Inc., San Diego, CA, USA) that in addition to standard genome-wide tagging SNPs (n = ~240,000) and exomic variants (n = ~280,000) contained about 70,000 additional custom content variants. Imputation of the Michigan CoreExome Array genotyped data was performed using the Haplotype Reference Consortium with the Michigan Imputation Server. Variants with $R^2 \geq 0.3$ and minor allele frequency (MAF) $\geq$ 1% were selected, resulting in more than 17 million imputed variants with European ancestry after quality control and filtering.

Selecting European Ancestry

We used supervised learning mode in *Admixture* to calculate ancestry coefficients for our dataset.(Alexander et al., 2009) 1000 genome phase 3 data, which contains 2504 reference individuals (5 ancestries), were used as training samples in the

model. The 5 ancestry fractions (K_EUR, K_AFR, K_AMR, K_EAS, and K_SAS) are produced and we used a threshold of K_EUR > 0.9 to select the dataset of European ancestry.

Characterization and Quality Control

As part of the independent data flow for both individual sites, sample and genotype quality control included assessment of call rates, gender check, cryptic relatedness, SNP missingness and the Hardy-Weinberg equilibrium. The standardized, pre-determined quality control metrics of each respective Biobank were retained, leading to minor differences in quality control metrics between BioVU and MGI. BioVU excludes SNPs with overall call rate < 95%, high sample missingness (> 5%), or significant deviation from Hardy-Weinberg Equilibrium ($P < 10^{-6}$).(Bajaj et al., 2020) Michigan Genomics Initiative excludes SNPs with overall call rate < 99%, high sample missingness (>1%), or significant deviation from Hardy-Weinberg Equilibrium ($P < 10^{-4}$) within each array.(Fritsche et al., 2018; Zawistowski et al., 2021) Principal components (PC) were generated using the EIGENSTRAT method in EigenSoft version 7.2.1 program to control for population stratification. Additionally, the variants used for the polygenic risk score were selected from imputed data with $R^2 > 0.3$, hard call threshold ± 0.1, and minor allele frequency > 1%.(Choi et al., 2020) Only chromosomes 1-22 are included in the analysis.

References

Alexander, D. H., Novembre, J., and Lange, K. (2009). Fast model-based estimation of ancestry in unrelated individuals. *Genome Res.* 19, 1655–1664.

Bajaj, A., Ihegword, A., Qiu, C., Small, A. M., Wei, W.-Q., Bastarache, L., et al. (2020). Phenome-wide association analysis suggests the APOL1 linked disease spectrum primarily drives kidney-specific pathways. *Kidney Int.* 97, 1032–1041.

Choi, S. W., Mak, T. S.-H., and O'Reilly, P. F. (2020). Tutorial: a guide to performing polygenic risk score analyses. *Nat. Protoc.* 15, 2759–2772.

Fritsche, L. G., Gruber, S. B., Wu, Z., Schmidt, E. M., Zawistowski, M., Moser, S. E., et al. (2018). Association of Polygenic Risk Scores for Multiple Cancers in a Phenome-wide Study: Results from The Michigan Genomics Initiative. *Am. J. Hum. Genet.* 102, 1048–1061.

Zawistowski, M., Fritsche, L. G., Pandit, A., Vanderwerff, B., Patil, S., Schmidt, E. M., et al. (2021). The Michigan Genomics Initiative: a biobank linking genotypes and electronic clinical records in Michigan Medicine patients. *bioRxiv*. doi: 10.1101/2021.12.15.21267864.

TRIPOD Checklist: Prediction Model Development and Validation

Section/Topic	Item		Checklist Item	Page
**Title and abstract**				
Title	1	D;V	Identify the study as developing and/or validating a multivariable prediction model, the target population, and the outcome to be predicted.	NA
Abstract	2	D;V	Provide a summary of objectives, study design, setting, participants, sample size, predictors, outcome, statistical analysis, results, and conclusions.	5
**Introduction**				
Background and objectives	3a	D;V	Explain the medical context (including whether diagnostic or prognostic) and rationale for developing or validating the multivariable prediction model, including references to existing models.	8
	3b	D;V	Specify the objectives, including whether the study describes the development or validation of the model or both.	8
**Methods**				
Source of data	4a	D;V	Describe the study design or source of data (e.g., randomized trial, cohort, or registry data), separately for the development and validation data sets, if applicable.	9
	4b	D;V	Specify the key study dates, including start of accrual; end of accrual; and, if applicable, end of follow-up.	9
Participants	5a	D;V	Specify key elements of the study setting (e.g., primary care, secondary care, general population) including number and location of centres.	9
	5b	D;V	Describe eligibility criteria for participants.	9
	5c	D;V	Give details of treatments received, if relevant.	NA
Outcome	6a	D;V	Clearly define the outcome that is predicted by the prediction model, including how and when assessed.	10
	6b	D;V	Report any actions to blind assessment of the outcome to be predicted.	NA
Predictors	7a	D;V	Clearly define all predictors used in developing or validating the multivariable prediction model, including how and when they were measured.	11
	7b	D;V	Report any actions to blind assessment of predictors for the outcome and other predictors.	NA
Sample size	8	D;V	Explain how the study size was arrived at.	14
Missing data	9	D;V	Describe how missing data were handled (e.g., complete-case analysis, single imputation, multiple imputation) with details of any imputation method.	9
Statistical analysis methods	10a	D	Describe how predictors were handled in the analyses.	12
	10b	D	Specify type of model, all model-building procedures (including any predictor selection), and method for internal validation.	18
	10c	V	For validation, describe how the predictions were calculated.	NA
	10d	D;V	Specify all measures used to assess model performance and, if relevant, to compare multiple models.	18
	10e	V	Describe any model updating (e.g., recalibration) arising from the validation, if done.	NA
Risk groups	11	D;V	Provide details on how risk groups were created, if done.	NA
Development vs. validation	12	V	For validation, identify any differences from the development data in setting, eligibility criteria, outcome, and predictors.	NA
**Results**				
Participants	13a	D;V	Describe the flow of participants through the study, including the number of participants with and without the outcome and, if applicable, a summary of the follow-up time. A diagram may be helpful.	16
	13b	D;V	Describe the characteristics of the participants (basic demographics, clinical features, available predictors), including the number of participants with missing data for predictors and outcome.	16
	13c	V	For validation, show a comparison with the development data of the distribution of important variables (demographics, predictors and outcome).	16
Model development	14a	D	Specify the number of participants and outcome events in each analysis.	16
	14b	D	If done, report the unadjusted association between each candidate predictor and outcome.	16
Model specification	15a	D	Present the full prediction model to allow predictions for individuals (i.e., all regression coefficients, and model intercept or baseline survival at a given time point).	18
	15b	D	Explain how to the use the prediction model.	NA
Model performance	16	D;V	Report performance measures (with CIs) for the prediction model.	18
Model-updating	17	V	If done, report the results from any model updating (i.e., model specification, model performance).	NA
**Discussion**				
Limitations	18	D;V	Discuss any limitations of the study (such as nonrepresentative sample, few events per predictor, missing data).	22
Interpretation	19a	V	For validation, discuss the results with reference to performance in the development data, and any other validation data.	NA
	19b	D;V	Give an overall interpretation of the results, considering objectives, limitations, results from similar studies, and other relevant evidence.	22
Implications	20	D;V	Discuss the potential clinical use of the model and implications for future research.	24
**Other information**				
Supplementary information	21	D;V	Provide information about the availability of supplementary resources, such as study protocol, Web calculator, and data sets.	Supp
Funding	22	D;V	Give the source of funding and the role of the funders for the present study.	28

*Items relevant only to the development of a prediction model are denoted by D, items relevant only to a validation of a prediction model are denoted by V, and items relating to both are denoted D;V. Note: when the checklist is complete, please ensure that it is blinded with identifying information removed from the properties section of the document and upload it as a Reporting Checklist on the file upload page of ScholarOne Manuscripts. If it is not included with your manuscript, we will not consider the submission for publication in our journal. Information on the TRIPOD Initiative is available at http://www.tripod-statement.org/.
